# Targeting glutamine metabolism exhibits anti-tumor effects in thyroid cancer

**DOI:** 10.1007/s40618-023-02294-y

**Published:** 2024-02-22

**Authors:** G.-Q. Zhang, C. Xi, N.-T. Ju, C.-T. Shen, Z.-L. Qiu, H.-J. Song, Q.-Y. Luo

**Affiliations:** Department of Nuclear Medicine, Shanghai Sixth People’s Hospital Affiliated to Shanghai Jiao Tong University School of Medicine, 600 Yishan Road, Shanghai, 200233 People’s Republic of China

**Keywords:** Thyroid cancer, Metabolism reprogramming, Glutamine, DON, Treatment

## Abstract

**Background:**

Effective treatment for patients with advanced thyroid cancer is lacking. Metabolism reprogramming is required for cancer to undergo oncogenic transformation and rapid tumorigenic growth. Glutamine is frequently used by cancer cells for active bioenergetic and biosynthetic needs. This study aims to investigate whether targeting glutamine metabolism is a promising therapeutic strategy for thyroid cancer.

**Methods:**

The expression of glutaminase (GLS) and glutamate dehydrogenase (GDH) in thyroid cancer tissues was evaluated by immunohistochemistry, and glutamine metabolism-related genes were assessed using real time-qPCR and western blotting. The effects of glutamine metabolism inhibitor 6-diazo-5-oxo-l-norleucine (DON) on thyroid cancer cells were determined by CCK-8, clone formation assay, Edu incorporation assay, flow cytometry, and Transwell assay. The mechanistic study was performed by real time-qPCR, western blotting, Seahorse assay, and gas chromatography–mass spectrometer assay. The effect of DON prodrug (JHU-083) on thyroid cancer in vivo was assessed using xenograft tumor models in BALB/c nude mice.

**Results:**

GLS and GDH were over-expressed in thyroid cancer tissues, and GLS expression was positively associated with lymph-node metastasis and TNM stage. The growth of thyroid cancer cells was significantly inhibited when cultured in glutamine-free medium. Targeting glutamine metabolism with DON inhibited the proliferation of thyroid cancer cells. DON treatment did not promote apoptosis, but increased the proportion of cells in the S phase, accompanied by the decreased expression of cyclin-dependent kinase 2 and cyclin A. DON treatment also significantly inhibited the migration and invasion of thyroid cancer cells by reducing the expression of N-cadherin, Vimentin, matrix metalloproteinase-2, and matrix metalloproteinase-9. Non-essential amino acids, including proline, alanine, aspartate, asparagine, and glycine, were reduced in thyroid cancer cells treated with DON, which could explain the decrease of proteins involved in migration, invasion, and cell cycle. The efficacy and safety of DON prodrug (JHU-083) for thyroid cancer treatment were verified in a mouse model. In addition to suppressing the proliferation and metastasis potential of thyroid cancer in vivo, enhanced innate immune response was also observed in JHU-083-treated xenograft tumors as a result of decreased expression of cluster of differentiation 47 and programmed cell death ligand 1.

**Conclusions:**

Thyroid cancer exhibited enhanced glutamine metabolism, as evidenced by the glutamine dependence of thyroid cancer cells and high expression of multiple glutamine metabolism-related genes. Targeting glutamine metabolism with DON prodrug could be a promising therapeutic option for advanced thyroid cancer.

**Supplementary Information:**

The online version contains supplementary material available at 10.1007/s40618-023-02294-y.

## Background

Thyroid cancer is an indolent tumor with better overall survival than other cancer types [1, 2]. However, effective treatments for patients with radioiodine refractory differentiated thyroid cancer and anaplastic thyroid cancer (ATC), which account for the majority of thyroid cancer-related deaths, are lacking. Various cancer treatments, including multi-kinase inhibitors and targeted kinase inhibitors, have been explored over the past few decades with promising results [3, 4]. However, cancer cells are prone to developing resistance to kinase inhibitors. Therefore, it is important to identify new alternative therapies to improve the outcome for advanced thyroid cancer patients.

Cancer cells undergo metabolic reprogramming to meet the active bioenergetic and biosynthetic demands for uncontrolled cell growth, redox status, and cell signaling [5], which is critical for cell survival and tumor progression in a hypovascularized and nutrient-deficient tumor microenvironment. Aerobic glycolysis is the best characterized metabolic alteration in cancer, which means that even under non-hypoxic conditions, glucose is primarily converted to lactate via glycolysis in cancer cells rather than flowing into mitochondrial oxidative phosphorylation, known as the “Warburg effect” [6]. Glutamine is the most abundant circulating amino acid, and glutamine metabolism becomes even more important in cells under pathophysiological conditions. When cardiomyocyte is exposed to oxidative stress, glutaminolysis, mediated by glutaminase (GLS), is enhanced to produce more alpha-ketoglutarate (α-KG) to replenish the tricarboxylic acid (TCA) cycle, contributing to cardioprotective effects by maintaining adenosine triphosphate and glutathione levels [7]. In skeletal muscle cells, thyroid hormones induce glutamine deamination flux by up-regulating glutamine pyruvate transferase 2 (GPT2), which catalyzes the conversion of glutamate and pyruvate into α-KG and alanine, to prevent muscle loss during atrophy [8]. The role of amino acid metabolism in tumorigenesis and progression has recently been recognized, and glutamine addiction is observed in many cancer types [9]. It is acknowledged that glutamine contributes its carbon backbone to the TCA cycle anaplerosis in the condition that glucose is devoted to aerobic glycolysis in cancer cells [10]. Coupling activated glycolysis and glutamine-dependent TCA cycle anaplerosis, driven by oncogene cooperation, is a prominent metabolic phenotype in cancer, in which GPT2 is a critical enzyme facilitating the entry of glutamine carbon into the TCA cycle [11]. In addition, glutamine is required for enhanced hexosamine biosynthesis pathway flux in cancer cells to synthesize uridine diphosphate *N*-acetylglucosamine under the catalyzation of glutamine-fructose amidotransferase 1 [12]. Uridine diphosphate *N*-acetylglucosamine is an essential substrate for *N*-glycosylation of membrane and secretory proteins to maintain protein homeostasis and is also an important substrate for *O*-GlcNAcylation of intercellular oncogenic proteins to modulate cell signaling [12]. Moreover, glutamine-derived glutathione plays a key role in tightly controlling reactive oxygen species metabolism to maintain redox balance [13]. Thus, glutamine metabolism is critical for tumorigenesis and progression.

Targeting glutamine metabolism is a promising therapeutic intervention strategy for cancer treatment. It was reported that 6-diazo-5-oxo-l-norleucine (DON) could irreversibly inhibit glutamine-utilizing enzymes by competitively binding to the glutamine active site with covalent adducts [14]. DON exhibited strong anti-tumor effects in multiple experimental tumor models [15–17]. The recently developed DON prodrugs were designed to circulate in the plasma in an intact and inert form; the prodrugs are preferentially cleaved by tumor-enriched esterases and peptidases and release DON in the tumor microenvironment while remaining inactivated in non-tumor tissues to reduce damage to non-target tissues [18, 19]. Monotherapy of ethyl 2-(2-amino-4-methylpentanamido)-DON (JHU-083), a newly developed prodrug, showed marked efficacy in tumor growth suppression and improved survival in preclinical studies [20–24].

However, few studies have explored glutamine metabolism programming in thyroid cancer and the therapeutic efficacy of targeting glutamine metabolism using DON or JHU-083 in thyroid cancer has not been determined. Therefore, in this study, we aimed to investigate the role of glutamine metabolism in thyroid cancer progression and the effect of targeting glutamine metabolism in thyroid cancer.

## Materials and methods

### Human samples, cell lines, and reagents

Thyroid cancer (CA) tissues and para-cancer (PA) tissues involved in this study were collected at Shanghai Sixth People’s Hospital Affiliated to Shanghai Jiao Tong University School of Medicine, with protocol approved by its Ethics Committee. Written consent has been obtained from each patient after a full explanation of the purpose and nature of all procedures used. Normal thyroid follicular epithelial cell line (Nthy-ori 3–1), papillary thyroid cancer (PTC) cell lines (TPC-1, K1, and BCPAP), follicular thyroid cancer (FTC) cell line (FTC-133), and ATC cell line (8305C) were obtained from Chinese Academy of Sciences (Shanghai, China). All cell lines were cultured in Roswell Park Memorial Institute (RPMI)-1640 medium (BIOAGRIO, China) supplemented with 10% fetal bovine serum (FBS, BIOAGRIO, China). RPMI-1640 glutamine-free medium (Gibco, USA) supplemented with 10% FBS (BIOAGRIO, China) was selected to determine the effect of glutamine deprivation on cell growth. DON, JHU-083, and bis-2-(5-phenylacetamido-1,2,4-thiadiazol-2-yl) ethyl sulfide (BPTES) were all purchased from MedChemExpress.

### Immunohistochemistry (IHC)

Paraffin-embedded CA and PA samples were collected to construct tissue microarrays (TMAs). IHC was performed on the TMAs with primary antibodies against GLS (Abcam, ab156876) and glutamate dehydrogenase (GDH, CST, #12793). The stained TMAs were scanned with an automatic digital slide scanner (Pannoramic MIDI, 3DHISTECH, Hungary) and were analyzed with supporting analysis software Alpathwell v2 (Servicebio, China). The staining intensity was scored as 0 (no staining), 1 (weak staining), 2 (moderate staining), or 3 (strong staining). The proportion score of positive cells was defined as (0, < 5%; 1, 6–25%; 2, 26–50%; 3, 51–75%; and 4, > 75%). Immunoreactivity score (IRS) was calculated by multiplying the staining intensity by the proportion score of positive cells. IRS of 4 was defined as a threshold dividing patients into the low- (< 4) and high-expression groups (≥ 4). As for IHC staining for xenograft tumor models, tumor tissues were sliced into 4.0 μm thickness. After deparaffinization, hydration, and antigen retrieval, tissue slides were incubated with primary antibodies against Ki-67 (Abcam, ab279653), cluster of differentiation 47 (CD47, Abcam, ab218810), programmed cell death ligand 1 (PD-L1, Abcam, ab213524), cluster of differentiation 206 (CD206, CST, #24595), and cluster of differentiation 86 (CD86, CST, #19589). After secondary antibodies incubation and nuclear staining, tissue slides were imaged under microscopy (Nikon Ts2, Japan).

### Cell viability, EdU assay, and colony formation assays

Cells (2×10^3^ for TPC-1 and K1, 5×10^3^ for BCPAP, FTC-133, and 8305C) were seeded in 96-well plates and were then cultured in RPMI-1640 medium, RPMI-1640 glutamine-free medium, RPMI-1640 medium with DON, or RPMI-1640 medium with BPTES. Cell Counting Kit-8 (CCK-8, Yeasen, Shanghai, China) assay was used to evaluate cell viability according to the manufacturer’s instructions. Cells (1×10^4^) were seeded in 24-well plates and were then cultured for 48 h in RPMI-1640 medium, RPMI-1640 glutamine-free medium, or RPMI-1640 medium with DON (0.5 μM). DNA synthesis was then evaluated by EdU Incorporation (Beyotime, Shanghai, China) according to the manufacturer’s instructions. For colony formation assay, cells (500 cells per well) were seeded in a 6-well plate. After incubation for 2 h, the supernatant was replaced by glutamine-free medium, or medium with DON (0.5 μM, 2.0 μM) or medium with BPTES (5.0 μM). Cells were cultured for 5 days (TPC-1), 7 days (K1), and 10 days (BCPAP and FTC-133) before crystal violet staining and imaging.

### Western blotting

To investigate the effect of DON on the expression of the targeted protein in thyroid cancer cells in vitro, TPC-1 and K1 cells were treated with phosphate-buffered saline (PBS) or DON (0.5 μM) for 48 h. Cells were then lysed using RIPA (Beyotime, Shanghai, China) with PMSF (1 mM, Beyotime, Shanghai, China), and the supernatants were collected after being centrifuged for 10 min at 4 degrees Celsius. As for the in vivo study, mice were all sacrificed at the end of the experiment, and tumor tissues were harvested. Tumor tissues were split into small pieces and were then ground with liquid nitrogen. Samples were lysed with RIPA, and the supernatants were collected after centrifugation. The primary antibodies involved are presented in Table S1.

### Real time-quantitative polymerase chain reaction (Rt-qPCR)

Cells (1×10^5^) were seeded in 6-well plates and cultured for 48 h. As for DON-treated cells, TPC-1 and K1 cells were cultured in medium with PBS or DON (0.5 μM) for 48 h. Total RNAs were isolated using an RNA extraction kit (AC0205, SparkJade, China), and 1 μg of RNA was used for reverse transcription with ABScript II cDNA First Strand Synthesis Kit (RK20400, ABclonal, China). Rt-qPCR was performed with SYBR green PCR mix (RK21203, ABclonal, China), and mRNA expression was normalized by the expression of actin and analyzed based on the relative quantification method 2^−ΔΔCt^. The primers involved are presented in Table S2.

### Transwell assay

Cells were treated with PBS or DON (0.5 μM) for 48 h. After being trypsinized and centrifuged, cells were then resuspended in FBS-free medium. For migration capacity assay, 1×10^4^ cells supported in FBS-free medium were seeded in Transwell chambers (8.0 μm pore size, Corning, USA), with 10% FBS medium in the lower wells. After incubation for 24 h, the upper cells were fixed with 4% paraformaldehyde and stained with crystal violet. For invasion capacity assay, 2×10^4^ TPC-1 cells or 1×10^4^ K1 cells were seeded in Transwell chambers with matrigel-precoated (356,234, Corning, USA), with the remaining operations same as migration assay. The stained cells were imaged using microscopy (Nikon Ts2, Japan), and analyzed using software Image J.

### Seahorse assay

To determine the effects of DON on oxidation phosphorylation and glycolysis, Seahorse assay was performed to quantitatively measure the oxygen consumption rate (OCR) and extracellular acidification rate assay (ECAR). Briefly, cells were pre-treated with PBS or DON (0.5 μM) for 48 h. For OCR detection, 1×10^4^ cells were cultured into a Seahorse XF 24-well microplate and their baseline measures were determined. Subsequently, cells were treated sequentially with oligomycin (1 μM), carbonyl cyanide 4-(trifluoromethoxy)phenylhydrazone (1 μM), and rotenone/antimycin A (1 μM) at indicated time points for measurement of OCR. Similarly, for ECAR detection, 5×10^3^ cells were cultured into a Seahorse XF 24-well microplate and treated sequentially with glucose (10 mM), oligomycin (1 μM), and 2-DG (50 mM) at indicated time points for measurement of ECAR. The assays were run on an Agilent Seahorse XFe24. OCR was reported as pmol/min and ECAR was reported as mpH/min.

### Flow cytometry analysis

For cell cycle analysis, cells (1×10^5^) were seeded in 6-well plates and treated with PBS or DON (0.5 μM) for 48 h. Cells were then trypsinized and centrifuged at 1000 rpm for 5 min. Cell cycle was assessed using a detection kit (Beyotime, Shanghai, China) according to the manufacturer’s instructions. Cell cycle was analyzed using a flow cytometer (Beckman Coulter, USA). The effect of DON on the apoptosis of thyroid cancer cells was evaluated using a detection kit (Biolegend, USA) according to the manufacturer’s instructions. Briefly, cells (1×10^5^) were incubated with PBS or DON (0.5 μM) for 48 h and harvested with trypsin. After two washes with PBS, cells were double-stained with APC-Annexin V/7-AAD for 15 min at room temperature. After that, apoptotic cells were analyzed using a flow cytometer (Beckman Coulter, USA).

### Gas chromatography–mass spectrometer assay

Gas chromatography–mass spectrometer analysis was conducted to measure the changes in cellular metabolites upon DON treatment. Cells were cultured in medium with PBS or DON (0.5 μM) for 48 h. 1×10^7^ cells were collected using a scraper in a 1.5 ml Eppendorf tube, adding 1.0 mL of pre-cooled methanol:water (vol:vol = 4:1) to each sample. Samples were then transferred to a 4 mL glass vial. After adding 200 μL of chloroform to each aliquot, an ultrasonic homogenizer was employed to break up the cells for 6 min at 500w. All of the mixtures of each sample were transferred to 1.5 mL Eppendorf tubes, and 40 μL of L-2-chlorophenylalanine (0.06 mg/mL) dissolved in methanol was added as an internal standard. All samples were then extracted by ultrasonication for 20 min in ice-water bath. The extract was centrifuged at 4 °C (13,000 rpm) for 10 min. 400 μL of supernatant in a glass vial was dried in a freeze-concentration centrifugal dryer. And 80 μL of methoxylamine hydrochloride in pyridine (15 mg/mL) was subsequently added. The resultant mixture was vortexed vigorously for 2 min and incubated at 37 °C for 60 min. 50 μL of BSTFA (with 1% TMCS) and 20 μL n-hexane were added into the mixture, which was vortexed vigorously for 2 min and then derivatized at 70 °C for 60 min. The samples were placed at ambient temperature for 30 min before GC–MS analysis. QC sample was prepared by mixing aliquot of all samples to be a pooled sample. The derivatives samples were analyzed on an Agilent 7890B gas chromatography system coupled to an Agilent 5977B MSD system (Agilent Technologies Inc., CA, USA). An HP-5MS fused-silica capillary column (30 m × 0.25 mm × 0.25 μm, Agilent J & W Scientific, Folsom, CA, USA) was utilized to separate the derivatives. Helium (> 99.999%) was used as the carrier gas at a constant flow rate of 1 mL/min through the column. The injector temperature was maintained at 260 °C. Injection volume was 1 μL by split less mode. The initial oven temperature was 60 °C held at 60 °C for 0.5 min, ramped to 125 °C at a rate of 8 °C/min, to 210 °C at a rate of 8 °C/min, to 270 °C at a rate of 15 °C/min, to 305 °C at a rate of 20 °C/min, and finally held at 305 °C for 5 min. The temperature of MS quadrupole and ion source (electron impact) was set to 150 and 230 °C, respectively. The collision energy was 70 eV. Mass spectrometric data were acquired in a full-scan mode (m/z 50–500).

The obtained GC/MS data were imported into the software MS-DIAL, which performs peak detection, peak identification, MS2Dec deconvolution, characterization, peak alignment, wave filtering, and missing value interpolation. Metabolite characterization is based on the LUG database. A data matrix was derived. The three-dimensional matrix includes sample information, the name of the peak of each substance, retention time, retention index, mass-to-charge ratio, and signal intensity. In each sample, all peak signal intensities were segmented and normalized according to the internal standards with RSD greater than 0.3 after screening. After the data were normalized, redundancy removal and peak merging were conducted to obtain the data matrix. Finally, the relative abundance of metabolites in the NC and DON-treated groups was compared. For metabolite profiling experiments, the matrix was imported in R to carry out Principle Component Analysis to observe the overall distribution among the samples and the stability of the whole analysis process. Orthogonal Partial Least-Squares-Discriminant Analysis (OPLS-DA) and Partial Least-Squares-Discriminant Analysis were utilized to distinguish the metabolites that differ between groups. To prevent overfitting, sevenfold cross-validation and 200 Response Permutation Testing were used to evaluate the quality of the model. Variable Importance of projection values obtained from the OPLS-DA model was used to rank the overall contribution of each variable to group discrimination. A two-tailed Student’s T test was further used to verify whether the metabolites of difference between groups were significant. Differential metabolites were selected with VIP values greater than 1.0 and p values less than 0.05.

### Animal experiments

All animal procedures were approved by the Animal Ethics Committee of Shanghai Sixth People’s Hospital Affiliated to Shanghai Jiao Tong University School of Medicine. BALB/c nude mice (3–4 weeks) were selected for xenograft tumor models. 3×10^6^ cells of K1 in 100 μl PBS were injected subcutaneously in the right armpits of nude mice. After 1 week, mice were randomly divided into control and experiment groups (5 mice/group). Mice in the experiment group were first given oral JHU-083 (2 mg/kg) administration on days 8, 10, and 12. Tumor volumes were monitored before each dose and calculated using the formula (length×width^2^)/2. However, tumor volumes in the two groups showed similar growth after the third dose. To increase JHU-083 absorption, intraperitoneal administration (2 mg/kg) was then operated on days 15, 17, and 19. Mice were euthanized on day 22, and tumors were harvested and weighed. Other organs including the lung, small intestine, liver, and kidney were also collected for hematoxylin and eosin stain.

### Statistical analysis

All statistical analysis was performed using SPSS 26.0 software. Differences among groups were estimated using Student’s *t* test, one-way ANOVA, or Chi-squared test. All tests were two-sided, and *p* < 0.05 was considered statistically significant.

## Results

### Glutamine metabolism-related enzymes are highly expressed in thyroid cancer

**Fig. 1:**
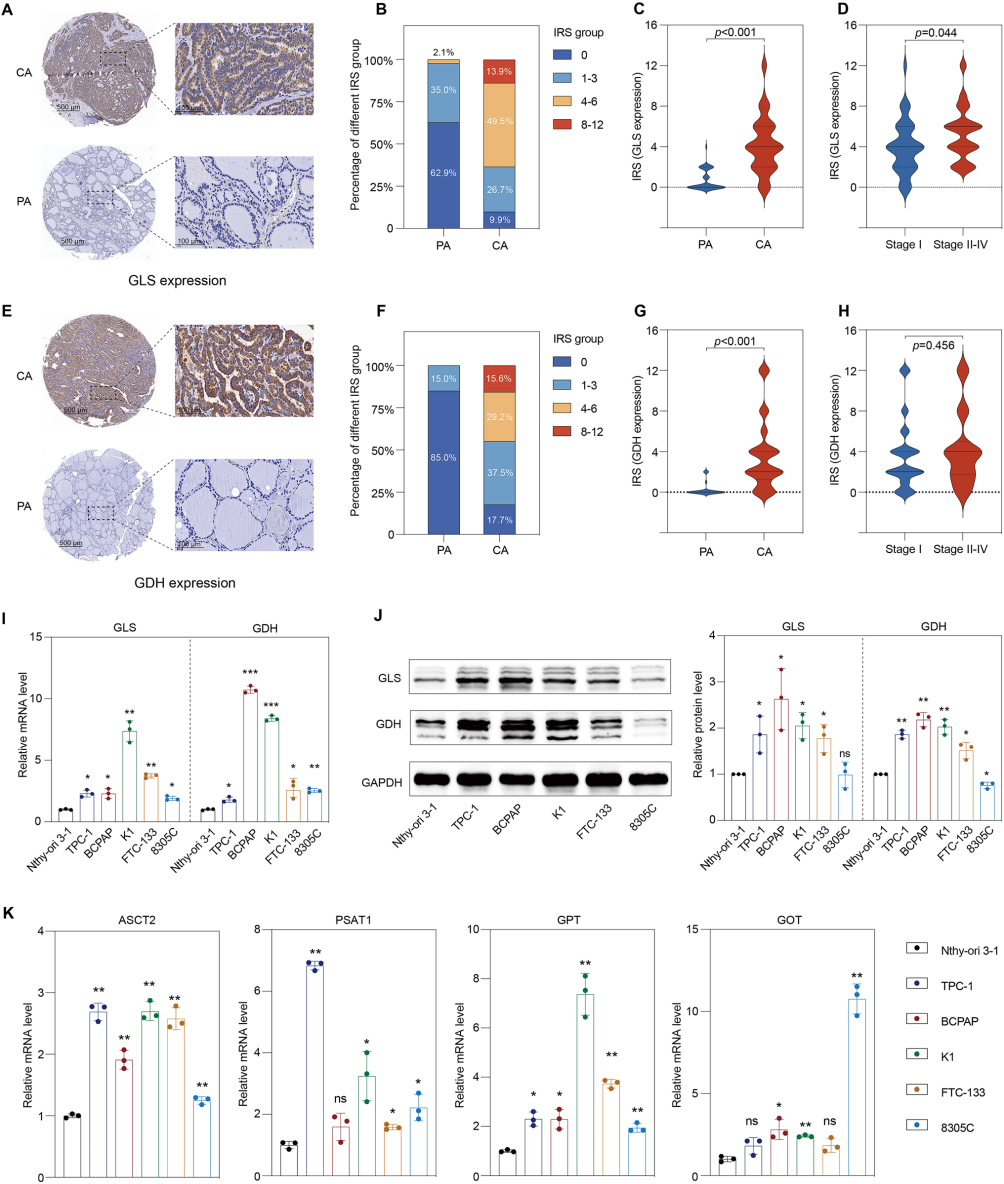
Glutamine metabolism-related enzymes are highly expressed in thyroid cancer. **A** Representative immunohistochemistry images of GLS expression in CA and PA specimens with low (40×) and high (400×) magnification. **B** Based on the IRS system on GLS expression, CA and PA specimens were divided into different score groups, with the percentage of each group presented. **C** The expression of GLS in CA samples was significantly higher than that in PA samples. **D** GLS expression was positively associated with the TNM stage. **E** Representative immunohistochemistry images of GDH expression in CA and PA specimens with low (40×) and high (400×) magnification. **F** Based on the IRS system on GDH expression, CA and PA specimens were divided into different score groups, with the percentage of each group presented. **G** The expression of GDH in CA samples was significantly higher than that in PA samples. **H** GDH was not significantly correlated with the TNM stage. **I** Rt-qPCR showed that the mRNA levels of GLS and GDH in thyroid cancer cell lines (TPC-1, BCPAP, K1, FTC-133, 8305C) were higher than those in normal thyroid follicular epithelial cells (Nthy-ori 3–1). **J** The protein levels of GLS and GDH in normal thyroid follicular epithelial cells and thyroid cancer cell lines. **K** The mRNA levels of ASCT2, PSAT1, GPT, and GOT in thyroid cancer cell lines. ASCT2, solute carrier family 1 neutral amino acid transporter member 5; CA, cancer; GDH, glutamate dehydrogenase; GLS, glutaminase; GOT, glutamic-oxaloacetic transaminase; GPT, glutamic-pyruvate transaminase; IRS, immune reactivity score; ns, not significant; PA, para-cancer; PSAT1, phosphoserine aminotransferase 1. *p < 0.05, **p < 0.01, ***p < 0.001

GLS and GDH convert glutamine to α-KG to fuel energy metabolism and biosynthesis, and GLS is the rate-limiting enzyme in glutaminolysis. We first evaluated GLS expression in thyroid cancer and para-cancer specimens using IHC; representative images are presented in Fig. 1A. Based on IRS, the percentage of cases in different score groups is displayed in Fig. 1B. More than half of the PA samples (88/140, 62.9%) had an IRS of 0, and the expression of GLS was significantly increased in CA specimens (*p* < 0.001, Fig. 1C). Clinicopathological data were available for 95 cases for further analysis. GLS expression was positively associated with the TNM stage (*p* = 0.044, Fig. 1D). Based on a threshold of 4, high GLS expression was observed in 62 patients (62/95, 65.3%), and low GLS expression was observed in 33 patients (33/95, 34.7%). GLS expression was associated with pathological type (*p* = 0.001), tumor size (*p* = 0.021), and lymph-node metastasis (*p* = 0.005) (Table 1). Other clinicopathological factors were not significantly correlated with GLS expression. GDH expression in thyroid cancer was also studied, with representative images shown in Fig. 1E. All PA tissues had an IRS score under 2 points, and most cases had a score of 0 (119/140, 85.0%, Fig. 1F). GDH was over-expressed in thyroid cancer (*p* < 0.001, Fig. 1G). Among the 89 patients with available clinicopathological data, GDH expression was not associated with the TNM stage (*p* = 0.456, Fig. 1H). Based on a threshold of 4, 47 (47/89, 52.8%) and 42 (42/89, 47.2%) patients exhibited low and high GDH expression, respectively. GDH expression tended to be associated with pathological type, with higher expression in PTC than in FTC, although the difference was not significant (*p* = 0.098) (Table 1). Other clinicopathological factors did not show significant correlations with GDH expression.

**Table 1:** The association between clinicopathological characteristics and expression of GLS or GDH in thyroid cancer

Characteristics	GLS expression			GDH expression		
	Low expression	High expression	*P* value	Low expression	High expression	*P* value
Age at diagnosis, years Mean** ± SD**	39 ± 13	41 ± 14	0.530	40 ± 14	42 ± 14	0.496
Sex						
Male	13 (46.4%)	15 (53.6%)	0.122	17 (60.7%)	11 (39.3%)	0.311
Female	20 (29.9%)	47 (70.1%)		30 (49.2%)	31 (50.8%)	
Pathological type						
PTC	22 (27.5%)	58 (72.5%)	0.001	38 (49.4%)	39 (50.6%)	0.098
FTC	11 (73.3%)	4 (26.7%)		9 (75.0%)	3 (25.0%)	
Tumor size, cm						
≤ 2	14 (24.1%)	44 (75.9%)	0.021	25 (46.3%)	29 (53.7%)	0.295
2.1—4	12 (48.0%)	13 (52.0%)		14 (60.9%)	9 (39.1%)	
≥ 4	7 (58.3%)	5 (41.7%)		8 (66.7%)	4 (33.3%)	
Extrathyroidal extension						
No	20 (31.3%)	44 (68.8%)	0.305	33 (54.1%)	28 (45.9%)	0.719
Yes	13 (41.9%)	18 (58.1%)		14 (50.0%)	14 (50.0%)	
Multifocality						
No	20 (40.0%)	30 (60.0%)	0.256	23 (53.5%)	20 (46.5%)	0.901
Yes	13 (28.9%)	32 (71.1%)		24 (52.2%)	22 (47.8%)	
Laterality						
Unilateral	20 (36.4%)	35 (63.6%)	0.696	24 (51.1%)	23 (48.9%)	0.727
Bilateral	13 (32.5%)	27 (67.5%)		23 (54.8%)	19 (45.2%)	
Tumor stage						
T1–T2	20 (30.3%)	46 (69.7%)	0.171	31 (50.0%)	31 (50.0%)	0.421
T3–T4	13 (44.8%)	16 (55.2%)		16 (59.3%)	11 (40.7%)	
Lymph-node metastasis						
N0	16 (59.3%)	11 (40.7%)	0.005	14 (56.0%)	11 (44.0%)	0.895
N1a	11 (28.2%)	28 (71.8%)		18 (50.0%)	18 (50.0%)	
N1b	6 (20.7%)	23 (79.3%)		15 (53.6%)	13 (46.4%)	
Distant metastasis						
No	33 (36.3%)	58 (63.7%)	0.294	46 (53.5%)	40 (46.5%)	0.600
Yes	0 (0%)	4 (100%)		1 (33.3%)	2 (66.7%)	

*GLS* glutaminase, *GDH* glutamate dehydrogenase, *SD* standard deviation, *PTC* papillary thyroid cancer, *FTC* follicular thyroid cancer

We next investigated the expression of GLS and GDH in thyroid cancer cells in vitro. As a result, the mRNA levels of GLS and GDH in normal thyroid follicular epithelial cells (Nthy-ori 3–1) were lower than those in thyroid cancer cells, including PTC cell lines (TPC-1, BCPAP and K1), an FTC cell line (FTC-133), and an ATC cell line (8305C) (Fig. 1I). The expression of these two proteins was also significantly higher in PTC and FTC cells than in Nthy-ori 3–1 cells (Fig. 1J). GLS and GDH were robustly expressed in PTC cells compared with FTC cells, which was consistent with the IHC results from tumor specimens. However, inconsistent with the Rt-qPCR results, there was no significant difference in GLS protein level between 8305C and Nthy-ori 3–1 cells (Fig. 1J). Furthermore, the expression of GDH in 8305C cells was slightly weaker than that in Nthy-ori 3–1 cells (Fig. 1J). Other glutamine metabolism-related genes, such as solute carrier family 1 neutral amino acid transporter member 5 (SLC1A5, also known as ASCT2), phosphoserine aminotransferase 1 (PAST1), GPT, and glutamic-oxaloacetic transaminase (GOT), were also highly expressed in thyroid cancer cells (Fig. 1K).

### Glutamine is required for the growth of thyroid cancer cells

**Fig. 2:**
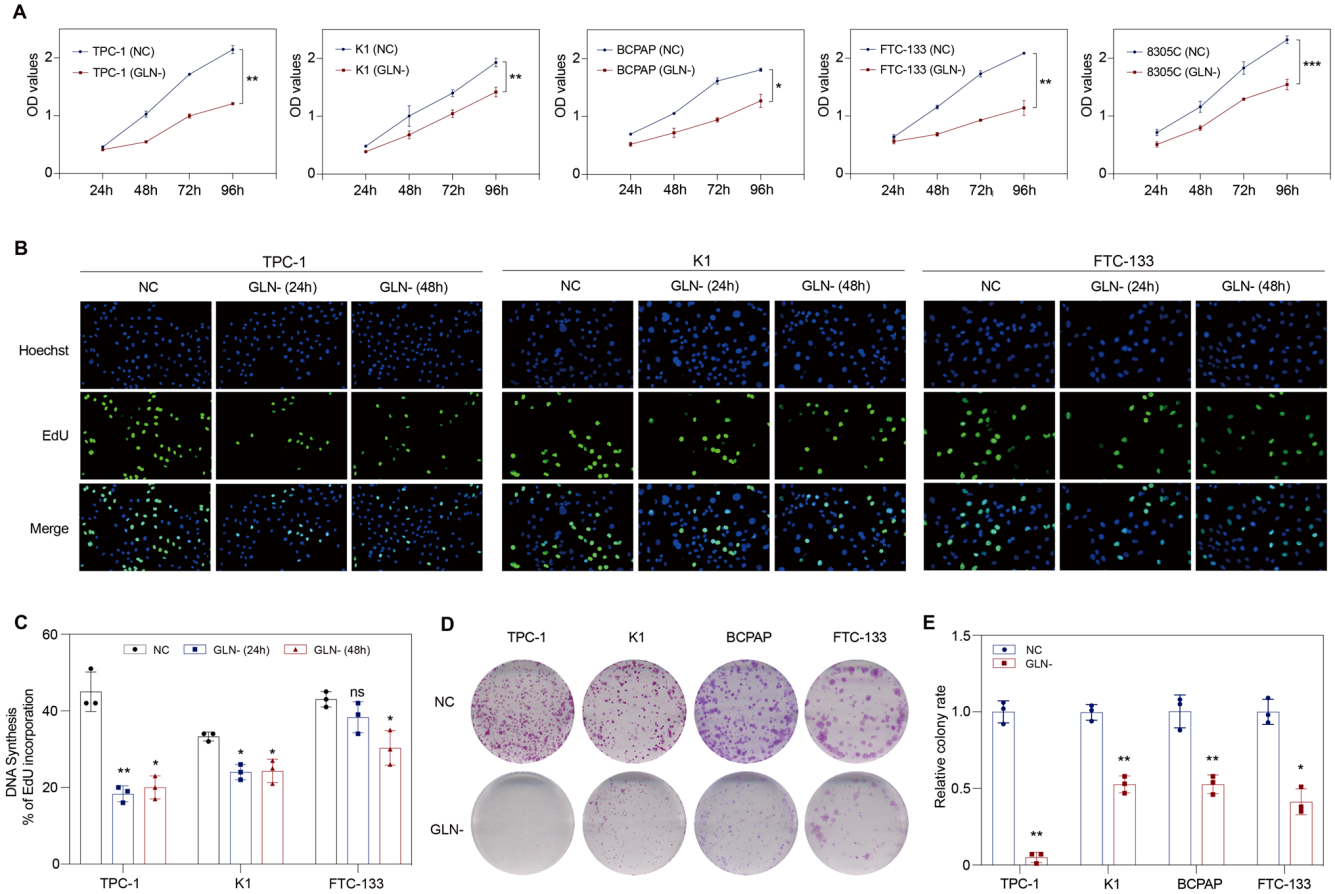
Glutamine is required for the growth of thyroid cancer cells. **A** CCK-8 assay demonstrated that cell viability significantly decreased upon glutamine deprivation. **B**–**C** EdU incorporation assay showed that DNA synthesis was reduced when cells were cultured in glutamine-free medium. **D**–**E** Glutamine deprivation significantly impaired the ability of thyroid cancer cells to form colonies. GLN-, glutamine-free; NC, normal control; ns, not significant. *p < 0.05, **p < 0.01, ***p < 0.001

CCK-8 assay was first used to determine the effect of short-term glutamine deprivation on cell viability. Glutamine deprivation remarkably inhibited cell viability in multiple thyroid cancer cell lines (Fig. 2A). DNA synthesis was dramatically reduced in TPC-1, K1, and FTC-133 cells when cultured in glutamine-free medium, and these results occurred as early as 24 h (Fig. 2B, C). Clone formation assay showed that long-term glutamine deprivation significantly impaired the ability of thyroid cancer cells to form clones (Fig. 2D, E). Collectively, these results suggest that thyroid cancer cells are dependent on glutamine.

### DON substantially causes growth suppression of thyroid cancer cells through inducing S-phase arrest

**Fig. 3:**
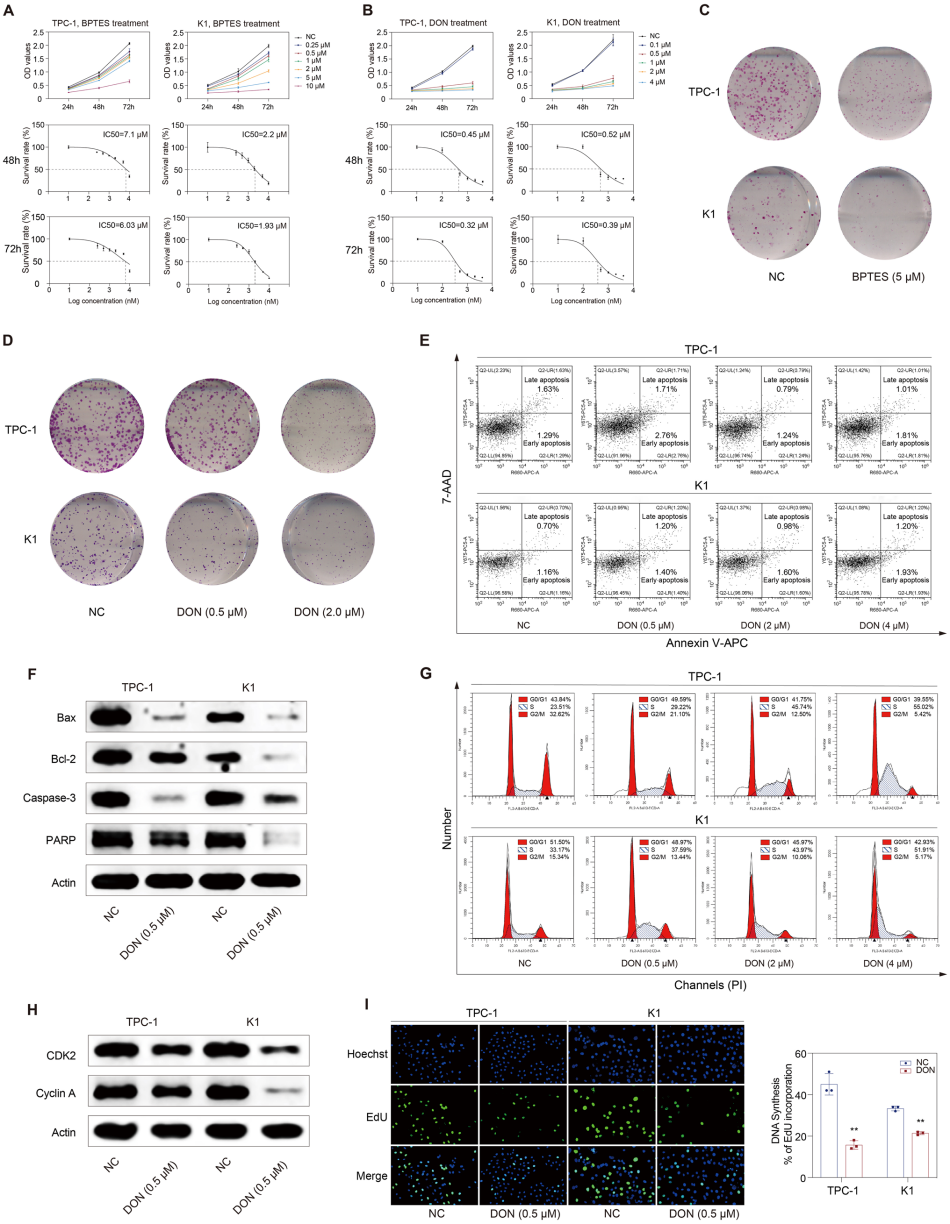
Targeting glutamine metabolism with DON suppresses the proliferation of thyroid cancer cells. **A** BPTES decreased the cell viability of thyroid cancer cells; the IC50 values of BPTES for 48 h in TPC-1 and K1 cells were 7.1 µM and 2.2 µM, respectively; the IC50 values of BPTES for 72 h in TPC-1 and K1 cells were 6.03 µM and 1.93 µM, respectively. **B** DON decreased the cell viability of thyroid cancer cells; the IC50 values of DON for 48 h for TPC-1 and K1 cells were 0.45 µM and 0.52 µM, respectively; the IC50 values of DON for 72 h for TPC-1 and K1 cells were 0.32 µM and 0.39 µM, respectively. **C**–**D** BPTES or DON impaired the clone formation capacity of TPC-1 and K1 cells. **E** Flow cytometry analysis showed that the proportion of apoptotic cells did not increase upon DON treatment. **F** Bax, Bcl-2, caspase-3, and PARP levels were decreased in response to DON treatment. **G** Flow cytometry analysis showed that DON markedly increased the proportion of tumor cells in the S phase in a dose-dependent manner. **H** The expression of CDK2 and cyclin A in TPC-1 and K1 cells was decreased after DON treatment. **I** EdU incorporation assay showed DON significantly inhibited DNA synthesis in TPC-1 and K1 cells. Bax, Bcl-2-associated X protein; Bcl-2, B-cell chronic lymphocytic leukemia-2; BPTES, bis-2-(5-phenylacetamido-1,2,4-thiadiazol-2-yl) ethyl sulfide; CDK2, cyclin-dependent kinase 2; DON, 6-diazo-5-oxo-l-norleucine; NC, normal control; PARP, poly ADP-ribose polymerase. **p < 0.01

Selective glutamine-utilizing enzyme inhibitors and broad-spectrum glutamine antagonists have been investigated to target glutamine metabolism in cancer. GLS is the rate-limiting enzyme for glutaminolysis, and its inhibitor BPTES exhibited remarkable tumor-killing effects in cancers [25, 26]. DON is a broadly active glutamine antagonist and could impede tumor growth of glioblastoma and pancreatic cancer [14–17]. Therefore, they were selected to examine the effects of targeting glutamine metabolism in thyroid cancer. Both BPTES and DON significantly decreased the cell viability of TPC-1 and K1 cells in time- and dose-dependent manners (Fig. 3A, B). BPTES or DON treatment also dramatically inhibited the clone formation capacity of thyroid cancer cells (Fig. 3C, D). Besides, we found that thyroid cancer cells were more sensitive to DON than to BPTES, so we subsequently focused on the treatment of DON in thyroid cancer.

Apoptosis is a common mechanism by which many treatments achieve anti-tumor effects. However, we did not find a remarkable change in the apoptosis rate of thyroid cancer cells after DON treatment (Fig. 3E). We also examined apoptosis-associated proteins and found that the expression of both anti-apoptotic protein B-cell chronic lymphocytic leukemia-2 (Bcl-2) and pro-apoptotic protein Bcl-2-associated X protein (Bax) decreased upon DON treatment (Fig. 3F). Similarly, caspase-3 and poly ADP-ribose polymerase (PARP) also decreased (Fig. 3F). These findings indicated that apoptosis was not the mechanism by which DON inhibited the growth of thyroid cancer cells. We subsequently evaluated the effect of DON on the cell cycle. The proportion of cells in the S phase was dramatically increased upon DON treatment in a dose-dependent manner, reaching more than 50% with 4 µM DON treatment (Fig. 3G). Cyclin A and cyclin-dependent kinase 2 (CDK2) are key factors that mediate the progression through the S phase, with cyclin A involved in the S-G2 transition [27]. Both protein levels were decreased after DON treatment (Fig. 3H), which suggested that DON induced S-phase arrest by down-regulating the expression of Cyclin A and CDK2. The hypothesis that DON caused growth suppression of thyroid cancer cells by interfering cell cycle was further supported by the results from EdU incorporation assay, which showed that DNA synthesis was significantly decreased upon DON treatment (Fig. 3I).

### DON treatment inhibits the migration and invasion of thyroid cancer cells

**Fig. 4:**
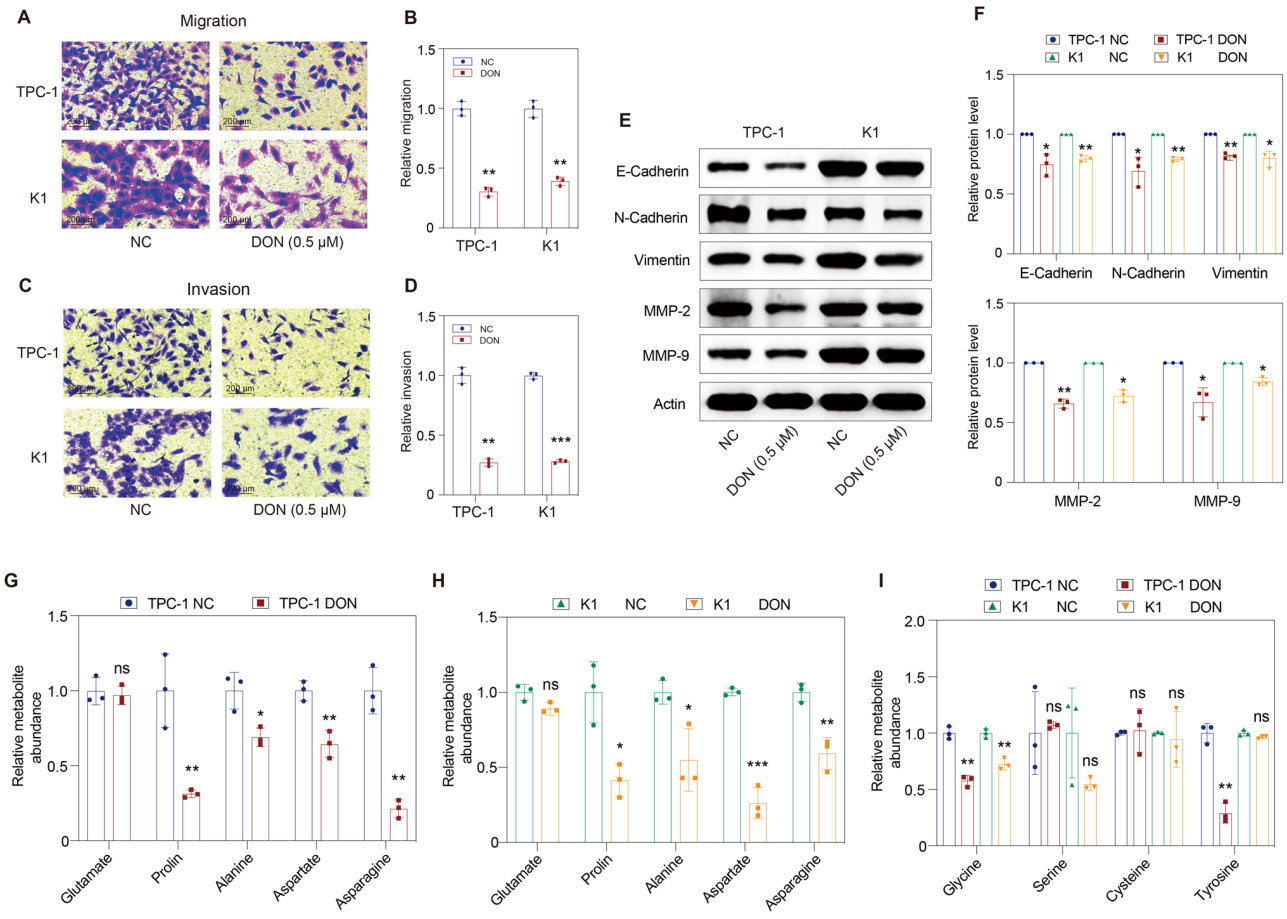
DON treatment significantly inhibits the migration and invasion of thyroid cancer cells. **A**–**B** The migration capacity of tumor cells was impaired by DON. **C**–**D** The invasion capacity of tumor cells was impaired by DON. **E**–**F** The expression of E-cadherin, N-cadherin, Vimentin, MMP-2, and MMP-9 was decreased upon DON treatment. **G**–**I** Some non-essential amino acids, including proline, alanine, aspartate, asparagine, and glycine, were decreased in TPC-1 and K1 cells upon DON treatment, while others, such as glutamate, serine, and cysteine, had no significant change. DON, 6-diazo-5-oxo-l-norleucine; MMP-2/9, matrix metalloproteinase-2/9; NC, normal control; ns, not significant. **p < 0.01, ***p < 0.001

Next, we evaluated the effect of DON on the migration and invasion ability of thyroid cancer. Transwell assay showed that the migration ability of TPC-1 and K1 cells was significantly decreased when cells were exposed to DON treatment (Fig. 4A, B). Similarly, the invasion ability was also remarkably impaired by DON treatment (Fig. 4C, D). It was suggested that DON treatment inhibited the metastatic potential of thyroid cancer cells. We initially speculated that DON achieved this success through inhibiting epithelial-interstitial transformation (EMT) of tumor cells. EMT inhibition is manifested by a decrease in mesenchymal cell markers N-cadherin and Vimentin and an increase in the epithelial cell–cell adhesion molecule E-cadherin [28]. We thus examined adhesion and cytoskeletal proteins involved in the EMT process and found that N-cadherin and Vimentin were decreased upon DON treatment (Fig. 4E, F). Unexpectedly, E-cadherin was also decreased in response to DON treatment (Fig. 4E, F). Additionally, the expression of matrix metalloproteinase (MMP)-2 and MMP-9 in tumor cells was decreased after DON treatment (Fig. 4E, F). These results suggested that DON could inhibit the metastatic potential of thyroid cancer cells with a mechanism other than EMT inhibition.

**Fig. 5:**
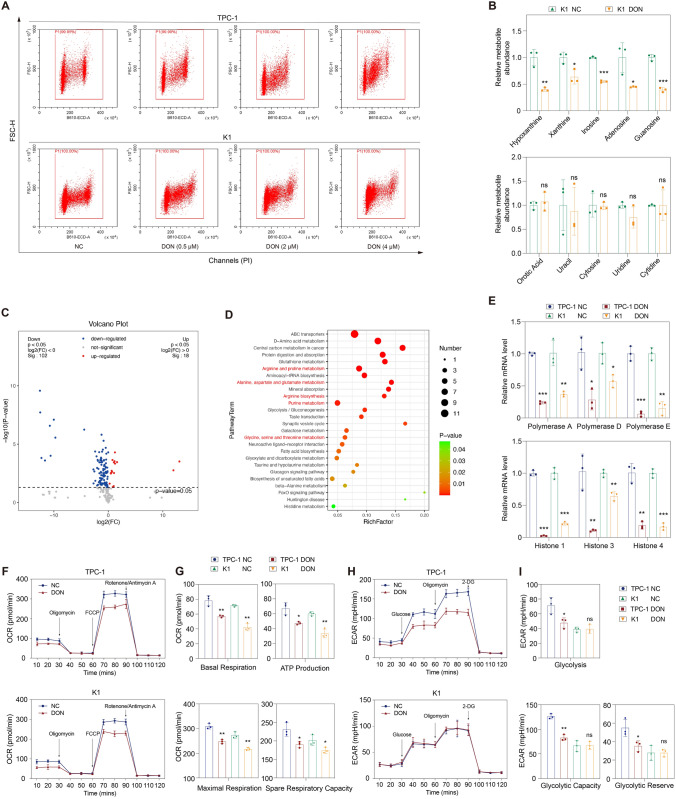
DON inhibits DNA synthesis and energy metabolism in thyroid cancer cells. **A** Flow cytometry analysis showed that, with the increase of DON concentration, the amount of DNA in the S-phase cells tended to be close to the haploid DNA (G1 phase) and away from the diploid DNA (G2/M phase). **B** Gas chromatography–mass spectrometer assay showed that hypoxanthine, xanthine, inosine, adenosine, and guanosine were decreased in DON-treated K1 cells. Orotic acid, uracil, cytosine, uridine, and cytidine were not affected by DON treatment. **C** Volcano plot of cellular metabolites in K1 cells significantly affected by DON. **D** KEGG pathway analysis of K1 cells with or without DON treatment. **E** Rt-qPCR showed that the mRNAs of DNA polymerases and histones were markedly decreased upon DON treatment. **F** OCR in TPC-1 and K1 cells with or without DON treatment. **G** Basal respiration, ATP production, maximal respiration, and spare respiratory capacity were significantly lower in DON-treated cells than in the NC cells. **H** ECAR assay in TPC-1 and K1 cells with or without DON treatment. **I** DON inhibited glycolysis, glycolytic capacity, and glycolytic reserve in TPC-1 cells, but not in K1 cells. DON, 6-diazo-5-oxo-l-norleucine; ECAR, extracellular acidification rate; NC, normal control; ns, not significant; OCR, oxygen consumption rate. *p < 0.05, **p < 0.01, ***p < 0.001

We noticed that all the target proteins tested above were reduced after DON treatment. It has been reported that at least 50% of non-essential amino acids (NEAAs) required for protein synthesis in cancer cells are derived from glutamine [29]. We next measured cellular NEAAs levels. Most of NEAAs, such as proline, alanine, aspartate, asparagine, and glycine, were decreased in TPC-1 and K1 cells treated with DON (Fig. 5G, H). Tyrosine decreased in TPC-1 cells treated with DON, while glutamate, serine, and cysteine remained unchanged in TPC-1 and K1 cells after DON treatment (Fig. 5G–I). NEAAs are required for macromolecule synthesis. We speculate that DON inhibits protein synthesis by disrupting cellular NEAAs levels, which could explain the decrease of proteins involved in migration, invasion, apoptosis, and cell cycle.

### DON inhibits DNA synthesis and energy metabolism in thyroid cancer cells

Flow cytometry showed that the amount of DNA in S-phase cells tended to be close to the haploid DNA (G1 phase) and away from the diploid DNA (G2/M phase) with the increase of DON concentration (Fig. 5A), suggesting that DON inhibited DNA synthesis in S-phase cells. We next attempted to uncover the mechanism by which DON interfered with DNA synthesis. Nitrogen in glutamine is an important element for the synthesis of nucleotide and nucleotide derivatives [30]. GC–MS assay was conducted and showed that hypoxanthine and xanthine, which are involved in the purine nucleotide biosynthesis pathway, were decreased in DON-treated K1 cells (Fig. 5B). Cellular adenosine, guanosine, and their precursor inosine were also decreased (Fig. 5B). Orotic acid, an intermediate in pyrimidine metabolism, showed no significant change in DON-treated cells (Fig. 5B). Similarly, uracil, cytosine, uridine, and cytidine, involved in pyrimidine nucleotide biosynthesis, did not exhibit significant reduction upon DON treatment (Fig. 5B). Metabolic profiling in K1 cell extracts with or without DON treatment was performed to further investigate metabolism alterations. Of the 245 detected metabolites, 102 metabolites were decreased and 18 metabolites were increased (Fig. 5C). KEGG enrichment analysis revealed that amino acid metabolism and purine metabolism were greatly affected by DON treatment (Fig. 5D). During the S phase, purine and pyrimidine nucleotides are used by DNA polymerases to produce DNA double helix, which envelops histones to form chromatin. We found that the mRNAs of DNA polymerases and histones in TPC-1 and K1 cells were also decreased upon DON treatment (Fig. 5E). These findings suggest that DON inhibits DNA synthesis in thyroid cancer by reducing substrates and related enzymes.

We also investigated the impact of DON treatment on energy metabolism in thyroid cancer cells. OCR, representing mitochondrial respiratory function and ATP production, was measured using Seahorse XF analysis. As depicted in Fig. 5F, G, basal respiration and ATP production were inhibited by DON in thyroid cancer cells. Maximal respiration and spare respiration capacity were also significantly decreased in DON-treated cells (Fig. 5F, G). ECAR is used to reflect the overall glycolytic flux. Our data showed that DON remarkably inhibited glycolysis, glycolytic capacity, and glycolytic reserve in TPC-1 cells but not in K1 cells (Fig. 5H, I). It is suggested that DON treatment impairs energy production in thyroid cancer cells by inhibiting oxidative phosphorylation and glycolysis.

### JHU-083 treatment results in tumor growth inhibition in vivo

**Fig. 6:**
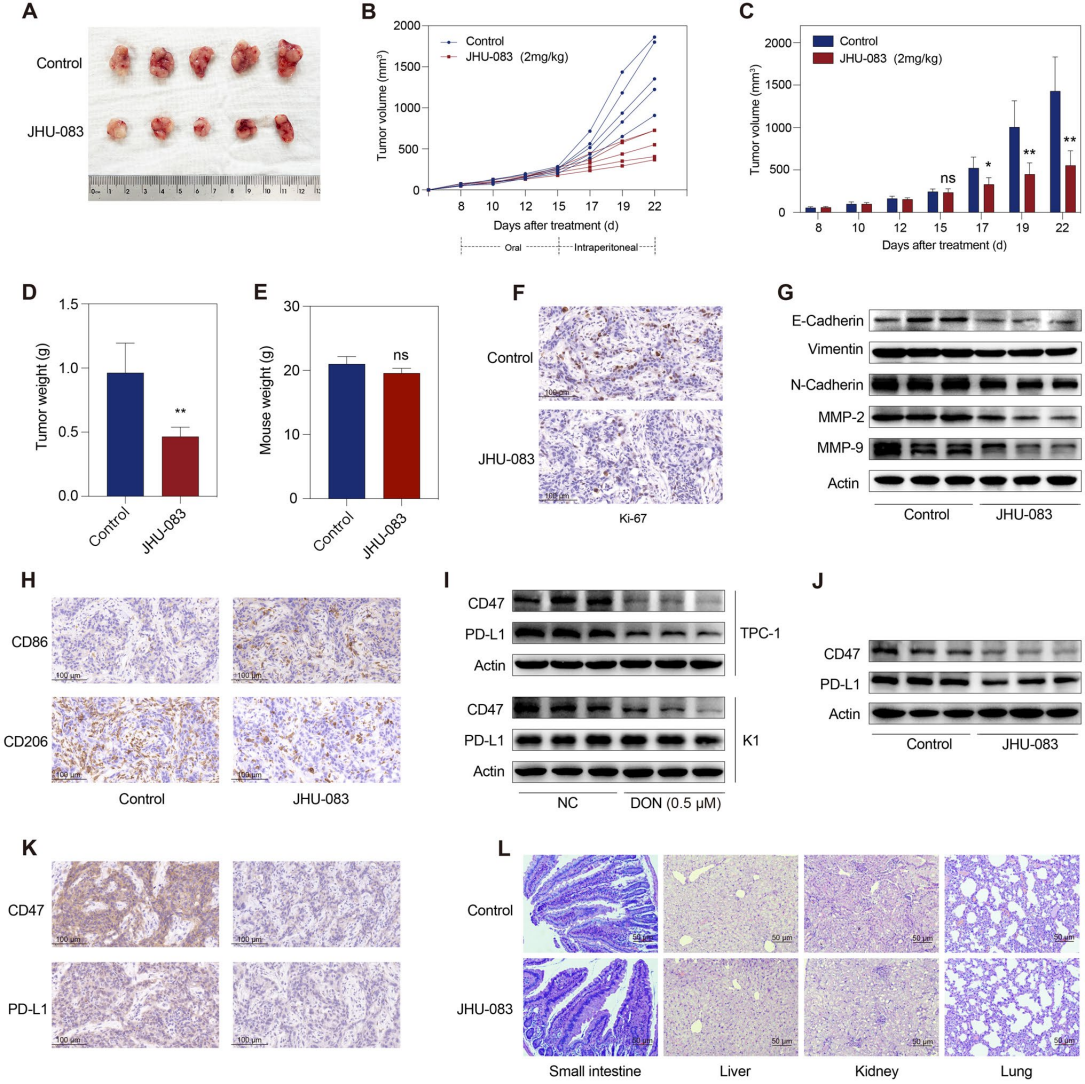
JHU-083 results in tumor growth inhibition in vivo. **A**–**C** K1 cells were selected to construct in vivo xenograft tumor models, and mice were treated with PBS or JHU-083 (5 mice/group) after tumor establishment. JHU-083 administration remarkably inhibited subcutaneous tumor growth. **D** Tumor weight in the JHU-083 group was significantly lower than that in the control group. **E** There was no significant difference in mouse weight between the two groups. **F** Ki-67-positive cells were fewer observed in tumor tissues with JHU-083 administration. **G** The expression of E-cadherin, vimentin, N-cadherin, MMP-2, and MMP-9 was decreased in the JHU-083 group. **H** CD206-positive cells were less distributed in the tumor stroma upon JHU-083 treatment, while CD86-positive cells were more distributed. **I** The expression of CD47 was decreased in TPC-1 and K1 cells upon DON treatment, while decreased PD-L1 expression was observed only in TPC-1 cells. **J** The expression of CD47 and PD-L1 was markedly decreased in the xenograft tumor model treated with JHU-083. **K** Immunohistochemistry images showed that the staining intensity of CD47 and PD-L1 in tumor cells weakened upon JHU-083 treatment. **L** Hematoxylin and eosin-stained images of small intestinal, liver, kidney, and lung tissues. CD47, cluster of differentiation 47; CD86, cluster of differentiation 86; CD206, cluster of differentiation 206; DON, 6-diazo-5-oxo-l-norleucine; JHU-083, monotherapy of ethyl 2-(2-amino-4-methylpentanamido)-DON; MMP-2/9, matrix metalloproteinase-2/9; NC, normal control; ns, not significant; PD-L1, programmed cell death ligand 1. *p < 0.05, **p < 0.01

We next used an in vivo xenograft tumor model to evaluate the anti-tumor effects of DON prodrug JHU-083 in thyroid cancer. As depicted in Fig. 6A–C, subcutaneous tumor growth was significantly inhibited by JHU-083. Tumor weight was remarkably reduced in the JHU-083 treatment group compared with the control group (Fig. 6D). There was no significant difference in mouse body weight between the treatment and control groups (Fig. 6E). Tumor tissues in the JHU-083 treatment group showed markedly decreased Ki-67-positive cells (Fig. 6F), indicating a loss of proliferative thyroid cancer cells. Furthermore, decreased protein levels of E-cadherin, Vimentin, N-cadherin, MMP-2, and MMP-9 were observed (Fig. 6G).

Previous study showed that DON and JHU-083 could increase the infiltration of activated macrophages and cytotoxic CD8^+^ T cells into tumor tissues to suppress tumor growth [16]. Immuno-compromised mouse was selected to construct an animal model in this study; therefore, the polarized states of tumor-associated macrophages (TAMs) were evaluated to determine whether JHU-083 treatment could enhance the innate immune response to thyroid cancer. M1 phenotype TAMs (M1-TAMs), as seen by CD86 staining, were more distributed in tumor tissues in the JHU-083 treatment group compared with the control group (Fig. 6H). There was a marked decrease of M2 phenotype TAMs (M2-TAM) in tumor tissues treated with JHU-083, as seen by CD206 staining (Fig. 6H). PD-L1 and programmed cell death receptor 1 are one pair of immune checkpoints that repress the innate and adaptive immune response to cancer cells [31, 32]. CD47 on the cancer cell membrane and its receptor signal-regulatory protein alpha on TAMs are another pair of immune checkpoints that inhibit the activation of macrophages against cancer [33]. Our data showed that CD47 was significantly down-regulated in TPC-1 and K1 cells after DON treatment in vitro (Fig. 6I). PD-L1 expression was decreased in DON-treated TPC-1 cells but not in K1 cells (Fg. 6I). In vivo results showed that the expression of CD47 and PD-L1 in thyroid cancer tissues was decreased in response to JHU-083 treatment (Fig. 6J, K). This suggests that JHU-083 could enhance the innate immune response to thyroid cancer through down-regulating immune checkpoints. These results also indicate that blocking glutamine utilization with JHU-083 may achieve anti-tumor effects by influencing the tumor microenvironment of thyroid cancer. Hematoxylin and eosin staining showed that no obvious damage to the structures of the small intestinal villus, liver, kidney, and lung was observed in JHU-083-treated mice (Fig. 6L).

## Discussion

In the present study, we found that thyroid cancer had enhanced glutamine metabolism, as evidenced by the glutamine dependence of thyroid cancer cells and high expression of multiple glutamine metabolism-related genes. Targeting glutamine metabolism with DON or JHU-083 markedly suppressed thyroid cancer growth. Mechanistically, DON caused attenuated cell proliferation in thyroid cancer by inducing S-phase arrest. The synthesis of proteins involved in promoting S-phase progression and metastasis was markedly inhibited by DON treatment. Besides, DNA synthesis was also decreased in thyroid cancer upon DON treatment. Furthermore, the innate immune response to thyroid cancer was enhanced upon JHU-083 treatment, with M1-TAMs accumulated in JHU-083-treated xenograft tumors.

Tumor cells undergo metabolic reprogramming during oncogenic transformation and rapid tumorigenic growth [34]. They rewire metabolic pathways to accommodate the increased demands for energy, reducing equivalents, and cellular biosynthesis [35]. The prevalence of abundant glutamine in the blood provides a readily available source of carbon and nitrogen to satisfy these needs. Recent studies have uncovered a pleiotropic role for glutamine as a versatile nutrient that participates in energy formation, redox homeostasis, macromolecular synthesis, and signaling in cancer cells [36]. Glutamine addiction has been observed in various cancer types [9], including thyroid cancer, as evidenced by increased levels of proteins related to glutamine metabolism in PTC specimens and results showing that glutamine deprivation impaired the proliferation of PTC cells [26, 37]. Consistently, our study showed that glutamine metabolism was enhanced in thyroid cancer. Glutamine metabolism-related genes, including amino acid transporter (ASCT2), amino-releasing enzymes (GLS and GDH), and aminotransferases (GPT, GOT, and PSAT1), were all up-regulated in thyroid cancer cells. High GLS expression in tumor tissues was positively correlated with lymph-node metastasis and TNM stage in thyroid cancer patients. Moreover, the proliferation of multiple thyroid cancer cell lines was decreased when cultured in glutamine-free medium.

Metabolism reprogramming contributes to tumor survival and progression but also introduces metabolic vulnerability. Compared with adjacent normal tissues, multiple cancer subtypes exhibit higher GLS expression, and high GLS expression correlates with tumor aggressiveness and poor prognosis [38]. Therefore, a series of inhibitors targeting GLS, such as BPTES and CB-839, were developed. Our results showed that the proliferation of thyroid cancer cells was repressed in response to BPTES. However, a previous study reported that targeting glutamine metabolism by a GLS inhibitor alone is insufficient for cancer therapy [39]. Objective response was not observed in a phase I clinical trial of CB-839 as a single agent in relapsed/refractory acute myelocytic leukemia [40]. DON, a broad-spectrum antagonist of glutamine utilization, inhibited tumor growth in preclinical studies and clinical trials [14]. However, unacceptable gastrointestinal toxicities hindered further clinical development [14]. DON prodrugs were recently developed, including JHU-083 and sirpiglenastat (also known as DRP-104). These prodrugs are intact and inert in plasma but are preferentially converted into DON in the tumor microenvironment by tumor-enriched hydrolases; notably, the prodrugs show promising anti-tumor effects and remarkably less activation in gastrointestinal tissues [17, 41, 42]. It has been reported that a broad-spectrum antagonist of glutamine is more effective in treating cancer than selective inhibition of a single glutamine metabolism-related enzyme [16]. Here, we found that thyroid cancer cells were more sensitive to DON than to the selective GLS inhibitor BPTES. The proliferation capacity of tumor cells was remarkably decreased by DON or JHU-083. S-phase arrest accounted for the decreased proliferation of thyroid cancer cells by DON treatment, which was consistent with a previous study [43]. The expression of CDK2 and cyclin A, two important factors promoting S-phase progression, was decreased in DON-treated cells, leading to the accumulation of cells in the S phase. Moreover, the amount of newly synthesized DNA in the S phase was decreased with the increase of DON concentration. DNA is synthesized by DNA polymerases with nucleotides as the building blocks. DON treatment remarkably decreased the levels of both purine nucleotides and DNA polymerases, suggesting that DON inhibits DNA synthesis by reducing substrates and down-regulating related catalytic enzymes. DON treatment also significantly impaired the metastasis activity of thyroid cancer cells, not likely from EMT reversal, as epithelial and mesenchymal markers, including E-cadherin, N-cadherin, and Vimentin, were all decreased upon DON or JHU-083 treatment in vitro or in vivo. Intercellular NEAAs, including proline, alanine, aspartate, asparagine, and glycine, were decreased in response to DON treatment. Energy production was also impaired upon DON treatment, which was indicated by the inhibition of oxidative phosphorylation and glycolysis. NEAAs are essential to protein synthesis and ATP is required for the activities of thyroid cancer cells. Therefore, DON decreases the metastatic potential of thyroid cancer through inhibiting the synthesis of metastasis-related proteins and energy generation.

Tumor cells undergo immune evasion to resist immune cell attack. Reversing the exhausted phenotype of immune cells is a potent strategy for tumor immunotherapy. Previous studies reported that DON prodrugs enhanced the killing effect of immune cells on tumor cells. DRP-104 increased tumor-infiltrating lymphocytes, and the increased CD8 + T cells showed a proliferating phenotype and decreased exhaustion markers [42]. Similarly, JHU-083 administration caused a marked increase in infiltrating CD8 + T cells; the cells were highly proliferative, robustly activated, less exhausted, and exhibited a less anergic phenotype [20, 23]. Except for the reactivation effects on adaptive immune response, DON also facilitates the innate immune response in the tumor microenvironment, as evidenced by the increased M1-TAMs in tumors with JHU-083 or DRP-104 treatment [20, 42]. Our data suggest that DON or JHU-083 down-regulated the expression of CD47 and PD-L1 in thyroid cancer, which was due to protein synthesis inhibition by DON. In vivo study showed that M1-TAMs were enriched in tumors with JHU-083 treatment along with the decrease of M2-TAMs. We speculate that macrophages polarize to the anti-tumor M1 phenotype upon JHU-083 treatment as a result of CD47 and PD-L1 signaling suppression. However, the effect of JHU-083 on the adaptive immune response in thyroid cancer needs to be further studied using an immuno-competent animal model.

In summary, our results showed that targeting glutamine metabolism with DON or DON prodrug inhibited the proliferation and metastasis of thyroid cancer by interfering with protein and DNA synthesis and remodeled the tumor microenvironment to enhance the innate immune response to thyroid cancer. A first-in-human clinical trial to characterize the safety and anti-tumor effect of the DON prodrug DRP-104 in patients with advanced solid tumors is currently ongoing (NCT04471415). Our data support the use of DON prodrugs in patients with advanced thyroid cancer.

### Supplementary Information

Below is the link to the electronic supplementary material.

Supplementary file1 (DOC 41 KB)

Supplementary file2 (DOC 48 KB)

## Data Availability

The datasets generated during and/or analysed during the current study are available from the corresponding author on reasonable request.
