# Supplementary material for: Targeting glutamine metabolism exhibits anti-tumor effects in thyroid cancer
Source: J Endocrinol Invest. 2024 Feb 22;47(8):1953–69. doi: 10.1007/s40618-023-02294-y (PMC11266413; doi:10.1007/s40618-023-02294-y)
Supplement: Supplementary file 1 — Supplementary file1 (DOC 41 KB) [file 40618_2023_2294_MOESM1_ESM.doc]

**Table S1: Primary antibodies used in western blotting in this study**

| Target protein | Abbreviation | Primary antibody | Dilution |
| --- | --- | --- | --- |
| Glyceraldehyde-3-phosphate dehydrogenase | GAPDH | Affinity, T0004 | 1:5000 |
| Actin |  | Affinity, T0022 | 1:5000 |
| Glutaminase | GLS | Abcam, ab156876 | 1:1000 |
| Glutamate Dehydrogenase 1/2 | GDH | CST, #12793 | 1:1000 |
| E-cadherin |  | Abcam, ab40772 | 1:1000 |
| N-cadherin |  | Abcam, ab76011 | 1:1000 |
| Vimentin |  | Abcam, ab92547 | 1:1000 |
| Matrix metalloproteinase-2 | MMP-2 | Abcam, ab92536 | 1:1000 |
| Matrix metalloproteinase-9 | MMP-9 | Abcam, ab76003 | 1:1000 |
| B-cell chronic lymphocytic leukemia-2 | Bcl-2 | Abcam, ab32124 | 1:1000 |
| Bcl-2-associated X protein | Bax | Abcam, ab32503 | 1:1000 |
| Caspase-3 |  | Abcam, ab32351 | 1:1000 |
| Poly ADP-ribose polymerase | PARP | CST, #9532 | 1:1000 |
| Cyclin dependent kinase 2 | CDK2 | Abcam, ab32147 | 1:1000 |
| Cyclin A |  | Abcam, ab181591 | 1:1000 |
| Cluster of differentiation 47 | CD47 | Abcam, ab218810 | 1:1000 |
| Programmed cell death ligand 1 | PD-L1 | Abcam, ab213524 | 1:1000 |
