# Supplementary material for: Targeting glutamine metabolism exhibits anti-tumor effects in thyroid cancer
Source: J Endocrinol Invest. 2024 Feb 22;47(8):1953–69. doi: 10.1007/s40618-023-02294-y (PMC11266413; doi:10.1007/s40618-023-02294-y)
Supplement: Supplementary file 2 — Supplementary file2 (DOC 48 KB) [file 40618_2023_2294_MOESM2_ESM.doc]

| **Table S2: qPCR primer sequence of genes involved in this study** | | | |
| --- | --- | --- | --- |
| Gene |  | Primer Sequences (5’-3’) | Nucleotide number |
| Actin | Forward | CACCCAGCACAATGAAGATCAAGAT | 25 |
|  | Reverse | CCAGTTTTTAAATCCTGAGTCAAGC | 25 |
| GLS | Forward | ACTTCTCAGGGCAGTTTGCTTTC | 23 |
|  | Reverse | TATCCAGAGGAGGAGACCAGCAC | 23 |
| GDH | Forward | AAGGGAGGTATCCGTTACAGCA | 22 |
|  | Reverse | TGGTGAACCTCCTTGTGATCTTTT | 24 |
| ASCT2 | Forward | CATCATCCTCGAAGCAGTCAAC | 22 |
|  | Reverse | AACTCAGGCTCTGTGCTTCTCG | 22 |
| PSAT1 | Forward | GGCTTGAAAGCAGGAAGGTGT | 21 |
|  | Reverse | AATACACGTAGGAGGCATCTGGG | 23 |
| GPT | Forward | TGCGCCAGGGTGTGAAGAA | 19 |
|  | Reverse | GGCATCGTCAGGGAAGTTGG | 20 |
| GOT | Forward | CAATGGCTGCAAGAAGTGAAAG | 22 |
|  | Reverse | CTTTAGCCCTGTGAAACAGAACA | 23 |
| Histone 1 | Forward | AGGCGCCGCTAAAGCTAAG | 19 |
|  | Reverse | CCTTCTTAGGGCTCTTCGCC | 20 |
| Histone 3 | Forward | GAGATCCGCCGTTATCAGAAG | 21 |
|  | Reverse | CCGTCAGAGAGACCACAGTAATCAC | 25 |
| Histone 4 | Forward | GGATGTTGTCTACGCGCTCA | 20 |
|  | Reverse | GCCTTTTATGGGTGAGCTGTT | 21 |
| Poly A | Forward | GGTAAAGCACGCAATAAAGACAAGA | 25 |
|  | Reverse | GTGGTGGAGTTATTTGAGGTGTCT | 24 |
| Poly D | Forward | CTCCGCTCCTACACGCTCAA | 20 |
|  | Reverse | GGTCTGGTCGTTCCCATTCTG | 21 |
| Poly E | Forward | AGTCAGTGGACGGATAAGATGG | 22 |
|  | Reverse | AACCCTTTCTGGTCGCAATGTA | 22 |
| qPCR, quantitative polymerase chain reaction; GAPDH, Glyceraldehyde-3-phosphate dehydrogenase; GLS, Glutaminase; GDH, Glutamate Dehydrogenase; ASCT2, Solute carrier family 1 neutral amino acid transporter member 5; PSAT1, phosphoserine aminotransferase 1; GPT, glutamic-pyruvate transaminase; GOT, glutamic-oxaloacetic transaminase; Poly, DNA polymerase. | | | |
